# Early Recurrence of Ovarian Cancer during Pregnancy after Primary Staging Surgery in the First Trimester

**DOI:** 10.1155/2020/1737061

**Published:** 2020-03-03

**Authors:** Munetoshi Akazawa, Kazunori Hashimoto

**Affiliations:** Department of Obstetrics and Gynecology, Tokyo Women's Medical University Medical Center East, Japan

## Abstract

Cancer during pregnancy is rare. However, even during pregnancy, there is the possibility of recurrence for the patients. We present the case of recurrence of ovarian cancer during pregnancy regardless of primary staging surgery performed in the first trimester of the same pregnancy. The patient was a 29-year-old woman who underwent fertility-sparing surgery at 15 weeks of pregnancy for ovarian cancer (mucinous adenocarcinoma, FIGO stage IC). Omitting adjuvant chemotherapy during pregnancy, we continued the prenatal checkups in the outpatient. At 31 weeks of gestation, massive ascites emerged and oliguria/anuria developed acutely. We performed emergent cesarean section, diagnosing acute kidney injury during pregnancy. On surgical finding, there were a number of 1 cm sized nodules in the small bowel wall and peritoneum. The infant was appropriate for gestational age without any abnormalities. Oliguria continued due to rapid accumulation of ascites in the early postpartum period. After two cycles of chemotherapy, ascites decreased gradually and the markers gradually decreased. However, after six courses of chemotherapy, she suddenly complained of nausea and anorexia. CT imaging showed cancerous ileus and ascites fluids. The patient chose palliative care. Even in the case of nonadvanced cancer, it has the potential to be an extremely aggressive malignancy under the irregular hormonal environment of pregnancy.

## 1. Introduction

Cancer during pregnancy is estimated to develop in one in 1000 pregnancies [1, 2]. Since more women are delaying childbearing, cancer is said to be diagnosed more often in pregnant women [3]. Even during pregnancy, there is the possibility of recurrence. We present the case of recurrence of ovarian cancer during pregnancy after primary staging surgery in the first trimester.

## 2. Case Presentation

We present a 29-year-old Asian woman who underwent fertility-sparing surgery at 15 weeks of pregnancy for ovarian cancer, later undergoing emergent cesarean section and exploratory laparotomy at 31 weeks of pregnancy for recurrence of ovarian cancer. She has provided written permission for the case to be reported. There was neither family history of cancer nor past medical history.

**Figure 1:**
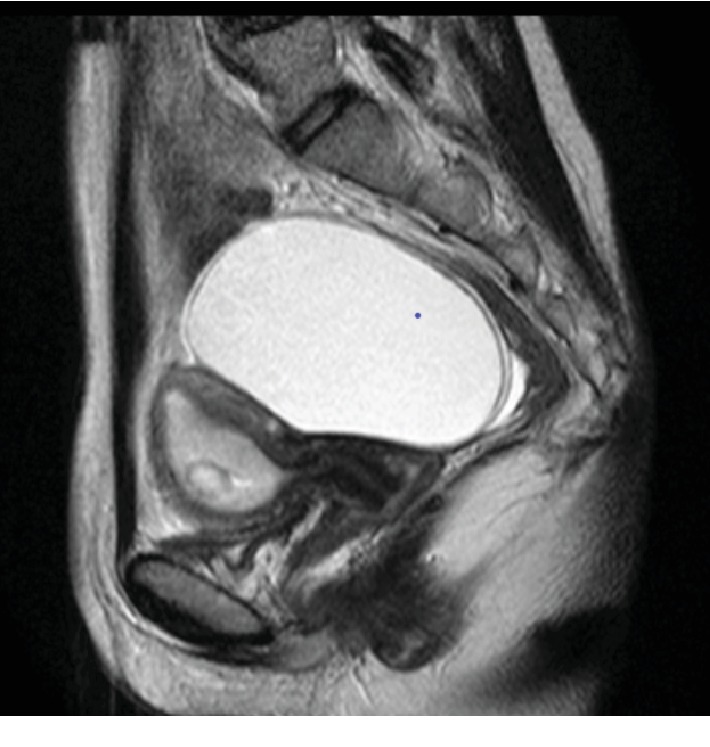
Sagittal T2-weighted MRI examination showed the possibility of benign ovarian cyst.

The patient, gravida 2, para 0, presented to her local obstetrician complaining of abdominal pain. On gynecologic examination, a 9 cm sized ovarian cyst was found by ultrasound examination. For surgical intervention, she was referred to a nearby hospital, where intrauterine pregnancy as well as ovarian cyst was found. Thus, she was referred to our hospital for the management of pregnancy complicated with an ovarian cyst. At the initial examination, sonographic evaluation suggested no malignancy: there was no ascites, Doppler ultrasound revealed no increased blood flow, and grayscale patterns appeared normal. Pelvic magnetic resonance imaging (MRI) examination also showed the possibility of benign ovarian cyst (Figure 1). The tumor markers were within normal limits (CA125, 27 U/mL; CA19-9, 17 U/mL). Considering the possibility of benign ovarian cyst, we continued regular prenatal checkup, setting surgical treatment at 15 weeks of pregnancy. In her first trimester of pregnancy, she had recurrent episodes of mild abdominal pain and vaginal bleeding, warranting admission.

**Figure 2:**
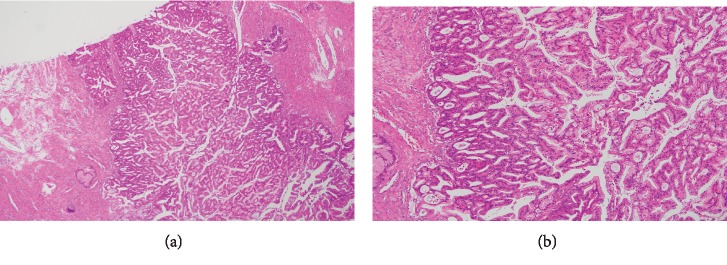
(a) Low-powered field view of hematoxylin eosin staining. (b) High-powered field view of hematoxylin eosin staining.

At 14 weeks and 0 days of gestation, the patient suddenly complained of severe left abdominal pain. On suspicion of torsion, the patient underwent emergent laparotomy. During surgery, 10 cm × 8 cm multiple left ovarian cysts and one rotation of the cyst were noted. With a diagnosis of torsion of ovarian cyst, we performed cystectomy of the left ovarian tumor. As a 1 cm sized small nodule on the wall of the specimen was noted, we performed careful exploration and washing cytology. The right ovary, uterus, and other intra-abdominal regions were normal. The postoperative course was uneventful. The final pathology was consistent with high-grade mucinous adenocarcinoma, endocervical type (Figures 2(a) and 2(b)). Washing cytology was negative. After a discussion involving the patient and her family, gynecologic oncologist, and maternal-fetal medicine specialist, secondary laparotomy as fertility-sparing surgery was selected.

At 15 weeks and 2 days of gestation, the patient underwent fertility-sparing surgery: exploratory laparotomy, left salpingo-oophorectomy, infracolic omentectomy, and cytologic examination of ascitic fluid. Intraoperative exploration showed a small amount of ascites and no residual lesion on the diaphragm, the surface of the liver, and Douglas' pouch. Lymph nodes were not palpated in the pelvis and para-aortic lesion. The postoperative course was uneventful except for postoperative ileus, treated with fasting and intravenous infusion from postoperative days 6-9. Final pathology showed no residual lesion on the omentum, and washing cytology was negative. A small atypical cell with large nuclei was found in the left residual ovary. Considering the fact that tumor was detected on the surface of the left residual ovary, the primary tumor stage was presumed to be ovarian cancer (FIGO stage IC2). After a repeated discussion, we omitted chemotherapy during pregnancy and continued the prenatal checkups in the outpatient department every 2 weeks.

At 31 weeks and 0 days of gestation, she called our department and complained of rapidly increasing abdominal circumference and nausea. Regular prenatal checkup findings 9 days before, including transvaginal ultrasound, were normal. Upon arrival, the patient appeared sick and ultrasound revealed massive ascites. Due to increasing dyspnea, paracentesis (approximately 3 L of ascitic fluid) was performed. Cytologic examination of ascetic fluid showed malignant cells. CA125 and CA19-9 levels were elevated by up to 317 U/mL and 3656 U/mL, respectively. Sooner after admission, oliguria/anuria developed acutely, which was caused by massive ascites and intravascular dehydration. Corticosteroids (two intramuscular injections of betamethasone of 12 mg 24 hours apart) were started to be administered for fetal lung maturity.

At 31 weeks and 3 days of gestation, blood urea nitrogen (BUN) and creatinine (Cr) were elevated due to uncontrolled anuria (BUN; 30.9 mg/dL, Cr: 1.12 mg/dL). Making a diagnosis of acute kidney injury, we performed emergent cesarean section. On surgical finding, there were a number of 1 cm sized nodules in the small bowel wall and <1 cm sized nodules in the abdominal wall and peritoneum. A total of 3.2 L of ascitic fluid was removed. There was no gross residual disease on the right ovary and appendix. The placenta and umbilical cord were normal, grossly and pathologically. Intraoperative biopsy confirmed metastatic ovarian carcinoma.

The infant was male and weighed 1422 g with an Apgar score of 8 and 9 at 1 and 5 min, respectively. The fetal blood counts were normal at the time of delivery. The infant showed minor acute respiratory distress syndrome and mild anemia but had no neurologic, psychomotor, or developmental abnormalities and was discharged 60 days after birth.

After cesarean section, the patient was transferred to the intensive care unit. Oliguria continued due to rapid accumulation of ascites in the early postpartum period. For management of malignant massive ascites, we repeated cell-free and concentrated ascites reinfusion therapy and maintained the amount of urine output. The baseline renal function tests improved gradually to the limit of normal on postoperative day 5. On postoperative 10 days, adjuvant chemotherapy consisting of paclitaxel (135 mg/m^2^) and carboplatin (the area under the curve: AUC, 5) was performed. In this period, the general condition of the patient was not good due to the massive ascites and anorexia. So, we chose the lower dosage of paclitaxel. Toxicity was evaluated according to the National Cancer Institute common toxicity criteria. The patient developed grade 3 neutropenia as a side effect. Meanwhile, after the first cycle of chemotherapy, she had pain and anorexia caused by massive ascites and abdominal bloating, which was controlled with total parenteral nutrition and continuous opioid infusion. After two cycles of chemotherapy, ascites decreased gradually and opioids were discontinued. Chemotherapy was repeated every 3 weeks, according to standard protocols. Two weeks after the first cycle of carboplatin/paclitaxel, tumor markers had increased to a peak value (CA125, 311 U/mL; CA19-9, 6095 U/mL). Thereafter, the markers gradually decreased. She tolerated the chemotherapy well and was discharged after three courses of chemotherapy. However, after six courses of chemotherapy, she suddenly complained of nausea and anorexia. CT imaging showed cancerous ileus and ascites fluids. After the discussion with the medical team, the patient chose palliative care.

## 3. Discussion

Cancer during pregnancy is estimated to develop in one in 1000 pregnancies [1, 2]. Since more women are delaying childbearing and the incidence of cancer in the 30 to 49 years age group is increasing, in the future, cancer is said to be diagnosed more often in pregnant women [3]. Cancer during pregnancy has been analyzed in several studies. de Haan reported 1170 cases of cancer during pregnancy in 20 years from multiple international institutions [4]. In this review, breast cancer was most common (39%), followed by cervical cancer (13%), lymphoma (10%), and ovarian cancer (7%). Of the patients, 88% had a live birth and 67% received treatment during pregnancy. The termination rate for the reason of cancer has decreased, and the rate of treatment during pregnancy has increased every 5 years in 20 years in this review. Chemotherapy during pregnancy was performed in 37% of patients. Regarding side effects of treatment during pregnancy, the relationship between platinum-based chemotherapy and small for gestation age, and the relationship between taxane chemotherapy and the increase of admission to neonatal intensive care unit admission, were suggested. In the case of cancer during pregnancy, the choice of the treatment is challenging, considering side effects of treatment against pregnancy and the infant.

Ovarian cancer during pregnancy has also been previously reviewed. In 2009, 41 cases were reviewed by Palmer et al. [5] and 105 cases by Blake in 2013 [6]. In the latter systematic review, the most common histology was serous adenocarcinoma (47%), followed by mucinous adenocarcinoma (27%), endometrioid adenocarcinoma (10%), and others. The majority of cases was stage I (63%), followed by stage III (24%). The live birth rate was 81%, and the most common nonviable pregnancy was elective termination (72%). Of the patients, 61% underwent fertility-sparing surgery and 36% received chemotherapy during pregnancy. Among 105 cases, 3 maternal deaths and 5 neonatal deaths were noted. The significance of chemotherapy and additional staging surgery during pregnancy was unclear in this study. So, decision of the treatment is so challenging during pregnancy. Palmer et al. suggested that the patient should be thoroughly counselled regarding to the chemotherapy [5]. Also, Blake et al. commented that the management of the cancer during pregnancy requires a multidisciplinary team [6]. Although the evidence is not enough regarding the treatment, thorough counselling with a multidisciplinary team is needed in this condition.

Similar clinical course of ovarian cancer during pregnancy was reported, although the case lacked the staging surgery in the first trimester of pregnancy [7]. In this case, a 25-year-old woman underwent emergent abdominal cystectomy for torsion of ovarian tumor at 6 weeks of gestation. The pathological diagnosis was mucinous adenocarcinoma, lacking the result of peritoneal washing cytology. Subsequent cytoreduction surgery and exploratory laparotomy were not performed by the patient's desire. At 39 weeks and 1 day of gestation, she complained of abdominal bloating. Emergent cesarean section and cytoreduction surgery were performed. Receiving six courses of chemotherapy against recurrence of ovarian cancer, she survived 5 years after surgery with no evidence of disease. The case was similar to our case in some points: the pathological type was mucinous adenocarcinoma; rapid accumulation of ascites was noted at recurrence during the third trimester of pregnancy; the response to chemotherapy was well regardless of mucinous type. The clinical course of the two cases was highly suggestive from the viewpoints of obstetrics and gynecologic oncology. From the obstetric standpoint, the intrauterine environment was not affected by the intra-abdominal condition. Massive nodules of metastatic cancer were sparse even on the uterus, and the fetus and placenta were not affected by cancer. From the standpoint of gynecologic oncology, the pregnancy seemed to affect a rapid course of recurrence. Regardless of the early stage of mucinous adenocarcinoma, the recurrence was highly aggressive; in contrast, response to chemotherapy was better. The atypical courses might be explained by the production of excessive endogenous sex and growth hormones during pregnancy [6].

Postoperative follow-up during pregnancy is unclear due to the rarity of the disease. Efficiency of tumor markers has been reported to be of limited value for substantiating a diagnosis during pregnancy because the serum CA125 levels may be elevated in normal pregnancy, usually peaking in the first trimester and returning to normal range in the second trimester [5]. However, CA125 levels increased to 381 U/mL in the case of relapse and 317 U/mL in our case. The fluctuation of CA125 levels in both cases suggested that CA125 may provide some information on ovarian cancer during pregnancy. As for imaging examination, computed tomography (CT) could not be used as screening test in pregnancy and ultrasound is an alternative evaluation tool. In the two cases, massive ascites developed rapidly at recurrence and relapse. Close examination of accumulation of ascites may lead to early diagnosis of recurrence.

Ovarian cancer during pregnancy is rare. Even in the case of nonadvanced cancer, it has the potential to be an extremely aggressive malignancy under the irregular hormonal environment of pregnancy. A high index of suspicion and close examination will be efficient in the management of the disease.
